# *Notes from the Field:* Methylmercury Toxicity from a Skin Lightening Cream Obtained from Mexico — California, 2019

**DOI:** 10.15585/mmwr.mm6850a4

**Published:** 2019-12-20

**Authors:** Anita Mudan, Lori Copan, Richard Wang, Amy Pugh, Jacob Lebin, Tracy Barreau, Robert L. Jones, Sutapa Ghosal, Mai Lee, Timothy Albertson, Jeffery M. Jarrett, Jean Lee, David Betting, Cynthia D. Ward, Alfonsina De Leon Salazar, Craig G. Smollin, Paul D. Blanc

**Affiliations:** ^1^California Poison Control System San Francisco Division; ^2^Department of Emergency Medicine, University of California, San Francisco; ^3^California Department of Public Health; ^4^Division of Laboratory Sciences, National Center for Environmental Health, CDC; ^5^Department of Medicine, University of California, San Francisco; ^6^Dignity Health Care System, Sacramento, California; ^7^California Poison Control System Sacramento Division; ^8^Department of Medicine, University of California, Davis; ^9^Department of Emergency Medicine, University of California, Davis; ^10^Division of Occupational and Environmental Medicine, Department of Medicine, University of California, San Francisco.

In July 2019, a Mexican-American woman aged 47 years in Sacramento, California, sought medical care for dysesthesias and weakness of her upper extremities. Over the ensuing 2 weeks of outpatient follow-up, her condition progressed to dysarthria, blurry vision, and gait unsteadiness, leading to hospital admission. While hospitalized, her condition declined rapidly to an agitated delirium. Two weeks into the hospitalization, screening blood and urine tests detected mercury concentrations exceeding the upper limit (UL) of quantification, indicative of abnormally high values of mercury (>160 *μ*g/L [blood] and >80 *μ*g/L [urine]). The hospital notified the California Poison Control System (CPCS) and the California Department of Public Health (CDPH). CPCS recommended oral dimercaptosuccinic acid, 10 mg/kg every 8 hours, which was administered via feeding tube. CDPH interviewed the patient’s family and learned that the patient was a long-term user of skin lightening creams obtained from Mexico (applied to the face twice daily for the past 7 years); the cream was analyzed and found to contain 12,000 ppm mercury. Mercury levels from the hospital specimens that initially implicated mercury were 2,620 *μ*g/L blood mercury (reference population UL <1.81 *μ*g/L)* and 110 *μ*g/L urine mercury (UL <0.90 *μ*g/L). A second blood specimen collected 11 days after the hospital initiation of ongoing dimercaptosuccinic acid chelation therapy detected 1,114 *μ*g/L mercury. 

The patient was transferred on hospital day 31 to a tertiary care facility, and a toxicology consultation was obtained the following day. Contaminated skin lightening creams typically contain inorganic mercury. Raman spectral analysis of the cream performed at CDPH, however, identified a possible match with methylmercury iodide, an organic mercury compound. Thus, organic mercury poisoning was suspected. The patient’s blood iodine level was 3,295 *μ*g/L (UL <92 *μ*g/L) at least 5 weeks after the last application of the cream. CDC confirmed values of blood total mercury 528 *μ*g/L, blood methyl mercury 460 *μ*g/L (UL <1.54 *μ*g/L), urine mercury 1,810 *μ*g/L, and urine iodine 20,100 *μ*g/L (UL <640 *μ*g/L)^†^ on specimens obtained 20 days after the initial specimen collections. The CDC assay for methyl mercury uses a reference method that does not differentiate it from methylmercury iodide (*1*). Despite prolonged chelation therapy, the patient remains unable to verbalize or care for herself, requiring ongoing tube feeding for nutritional support.

This is the first known case of contamination of skin lightening cream with methyl mercury (or any congener, including methylmercury iodide). In contrast, health risks associated with inorganic mercury exposure are well-recognized from such products; levels up to 200,000 ppm (typically mercurous chloride) have been reported (*2*,*3*). The relatively lower 12,000 ppm mercury content of the cream in this case underscores the far higher toxicity of organic mercury compounds. Central nervous system toxicity, the hallmark of organic mercury, typically manifests after weeks to months of exposure, progresses rapidly after onset, worsens despite cessation of further exposure, persists even with chelation (although mercury excretion might increase), and leaves profound residual impairment (*4*). In addition to methyl mercury, multiple congeners are toxic, including methylmercury iodide used in the synthesis of methyl mercury (*5*,*6*).

The original source of the methyl mercury adulterant and its marketing chain remain to be identified. CDPH is actively working to warn the public of this health risk, actively screening other skin lightening cream samples for mercury, and is investigating the case of a family member with likely exposure but less severe illness.

## Footnotes

* Upper limits for mercury based on the 95th percentile for the Mexican-American population in the National Health and Nutrition Examination Survey from 2015 to 2016. https://www.cdc.gov/exposurereport/index.html.

† Upper limit for urinary iodine based on the 95th percentile for the Mexican-American population in the National Health and Nutrition Examination Survey from 2005 to 2006. https://www.cdc.gov/nutritionreport/index.html.
